# Prevalence of Lung Cancer Screening in the US, 2022

**DOI:** 10.1001/jamanetworkopen.2024.3190

**Published:** 2024-03-21

**Authors:** Louise M. Henderson, I-Hsuan Su, M. Patricia Rivera, Joyce Pak, Xiaomeng Chen, Daniel S. Reuland, Jennifer L. Lund

**Affiliations:** 1 Department of Radiology, University of North Carolina at Chapel Hill; 2 Lineberger Comprehensive Cancer Center, University of North Carolina at Chapel Hill; 3 Department of Epidemiology, University of North Carolina at Chapel Hill; 4 Department of Medicine, University of Rochester Medical Center, Rochester, New York; 5 Department of Medicine, University of North Carolina at Chapel Hill

## Abstract

This cross-sectional study compares lung cancer screening prevalence in 2022 among individuals eligible by 2021 vs 2013 criteria by sociodemographics and state.

## Introduction

The National Lung Screening Trial reported a reduction in lung cancer mortality with low-dose computed tomography lung cancer screening (LCS) among individuals with significant smoking exposure. In 2013, the US Preventive Services Task Force (USPSTF) recommended annual LCS with low-dose computed tomography (CT) for individuals ages 55 to 80 years who currently or formerly smoked (quit within 15 years) with a 30 pack-year (PY) smoking history.^1^ In 2021, the USPSTF revised its recommendation to initiate LCS at age 50 years and lowered the smoking requirement to 20 PY, in part to address concerns over disparities in eligibility by race and ethnicity and sex.^2^ Despite recommendations, LCS uptake has been low, with 2021 data from 4 states reporting 21% of eligible individuals screened.^3^ National estimates of LCS prevalence are limited. Hence, we sought to compare LCS prevalence in 2022 by sociodemographic characteristics and by state among individuals eligible per 2013 vs 2021 USPSTF recommendations.

## Methods

The University of North Carolina at Chapel Hill institutional review board exempted this cross-sectional study from review and informed consent because data used are publicly available. We followed the STROBE reporting guideline.

We analyzed Center for Disease Control and Prevention Behavioral Risk Factor Surveillance System (BRFSS) data for 2022, which included LCS as a core section for all states.^4^ We included adults ages 50 to 79 years who self-reported currently smoking or quitting within the last 15 years with at least a 20-PY smoking history. Because USPSTF recommends yearly LCS, we classified responders who reported a chest CT scan to “check or screen for” lung cancer within the past year as having LCS per recommendations (Table).

**Table.** Characteristics of Individuals Eligible and Screened for Lung Cancer in 2022

Characteristic	2013 USPSTF criteria			2021 USPSTF criteria		
	Individuals, No. (%)		LCS prevalence (95% CI), %	Individuals, No. (%)		LCS prevalence (95% CI), %
	Eligible	Screened^a^		Eligible	Screened^a^	
Overall	8 154 440	1 598 865	19.6 (18.5-20.8)	13 526 348	2 217 919	16.4 (15.6-17.2)
Age, y						
50-54	NA	NA	NA	2 063 840 (15.3)	125 831 (5.7)	6.1 (5.0-7.2)
55-59	1 738 645 (21.3)	204 736 (12.8)	11.8 (10.2-13.4)	2 639 255 (19.5)	303 740 (13.7)	11.5 (10.1-12.9)
60-64	2 302 867 (28.2)	413 484 (25.9)	18.0 (16.4-19.5)	3 338 111 (24.7)	559 982 (25.2)	16.8 (15.3-18.3)
65-69	1 799 859 (22.1)	382 106 (23.9)	21.2 (19.5-23.0)	2 440 591 (18.0)	507 881 (22.9)	20.8 (19.1-22.5)
70-74	1 423 943 (17.5)	357 357 (22.4)	25.1 (22.2-28.0)	1 946 129 (14.4)	442 781 (20.0)	22.8 (20.4-25.1)
75-79	889 126 (10.9)	241 182 (15.1)	27.1 (23.8-30.5)	1 098 421 (8.1)	277 704 (12.5)	25.3 (22.3-28.3)
Median (IQR)	64.1 (59.6-69.8)	66.3 (61.4-71.6)	NA	62.1 (56.7-68.1)	65.2 (60.2-70.8)	NA
Race and ethnicity^b^						
Hispanic or Latino	345 218 (4.4)	78 596 (5.1)	22.8 (17.9-27.6)	641 116 (4.9)	100 884 (4.7)	15.7 (12.7-18.7)
Non-Hispanic						
American Indian or Alaska Native	191 099 (2.4)	27 883 (1.8)	14.6 (12.6-16.6)	308 728 (2.4)	39 881 (1.9)	12.9 (10.9-14.9)
Asian	93 387 (1.2)	37 341 (2.4)	40.0 (37.4-42.5)	175 228 (1.4)	40 419 (1.9)	23.1 (21.0-25.1)
Black	544 449 (6.9)	98 468 (6.4)	18.1 (14.4-21.8)	1 138 680 (8.8)	194 921 (9.1)	17.1 (14.3-19.9)
Native Hawaiian or Other Pacific Islander	27 674 (0.4)	2040 (0.1)	7.4 (4.5-10.2)	43 064 (0.3)	4184 (0.2)	9.7 (6.0-13.4)
White	6 626 844 (84.3)	1 301 241 (84.0)	19.6 (18.5-20.8)	10 617 578 (81.9)	1 755 951 (81.9)	16.5 (15.7-17.4)
Multiracial	28 283 (0.4)	3326 (0.2)	11.8 (8.3-15.2)	47 056 (0.4)	7038 (0.3)	15.0 (12.3-17.6)
Missing, No.^c^	297 486	49 971	NA	554 898	74 641	NA
Sex						
Male	4 680 912 (57.4)	912 066 (57.0)	19.5 (18.0-20.9)	7 348 909 (54.3)	1 223 562 (55.2)	16.6 (15.5-17.8)
Female	3 473 528 (42.6)	686 799 (43.0)	19.8 (18.1-21.5)	6 177 439 (45.7)	994 357 (44.8)	16.1 (14.9-17.3)
Smoking status						
Current	4 501 229 (55.2)	823 162 (51.5)	18.3 (16.8-19.8)	8 094 613 (59.8)	1 247 514 (56.2)	15.4 (14.4-16.4)
Former	3 653 211 (44.8)	775 703 (48.5)	21.2 (19.6-22.9)	5 431 735 (40.2)	970 405 (43.8)	17.9 (16.5-19.2)
Pack-years						
20-29	NA	NA	NA	4 020 879 (29.7)	528 665 (23.8)	13.1 (11.8-14.5)
30-39	2 140 976 (26.3)	307 693 (19.2)	14.4 (12.7-16.1)	2 969 351 (22.0)	345 579 (15.6)	11.6 (10.3-13.0)
≥40	6 013 464 (73.7)	1 291 172 (80.8)	21.5 (20.1-22.9)	6 536 118 (48.3)	1 343 675 (60.6)	20.6 (19.2-21.9)
Median (IQR)	46.5 (38.6-58.2)	49.3 (41.4-62.8)	NA	38.3 (26.9-50.6)	44.1 (29.7-55.5)	NA
Time since quit, y						
Currently smoke	4 501 229 (55.2)	823 162 (51.5)	18.3 (16.8-19.8)	8 094 613 (59.8)	1 247 514 (56.2)	15.4 (14.4-16.4)
<1	290 694 (3.6)	75 233 (4.7)	25.9 (23.0-28.8)	446 400 (3.3)	96 305 (4.3)	21.6 (19.2-24.0)
1-5	990 166 (12.1)	227 798 (14.2)	23.0 (20.4-25.6)	1 477 144 (10.9)	292 817 (13.2)	19.8 (17.5-22.1)
6-10	912 709 (14.7)	250 299 (15.7)	20.9 (18.1-23.7)	1 825 987 (13.5)	317 186 (14.3)	17.4 (15.1-19.7)
11-15	1 176 994 (14.4)	222 374 (13.9)	18.9 (16.8-21.0)	1 682 205 (12.4)	264 097 (11.9)	15.7 (14.0-17.4)
Insurance status						
Insured	7 531 089 (95.1)	1 540 329 (98.9)	20.5 (19.2-21.7)	12 367 483 (94.5)	2 128 345 (98.9)	17.2 (16.3-18.1)
Uninsured	386 771 (4.9)	16 683 (1.1)	4.3 (2.6-6.1)	715 854 (5.5)	24 178 (1.1)	3.4 (2.1-4.6)
Missing, No.^c^	236 580	41 853	NA	443 011	65 397	NA
Have health care clinician						
Yes	7 345 658 (90.7)	1 548 300 (97.3)	21.1 (19.9-22.3)	12 035 165 (89.7)	2 143 276 (97.1)	17.8 (16.9-18.7)
No	754 770 (9.3)	42 212 (2.7)	5.6 (4.2-7.0)	1 386 340 (10.3)	64 954 (2.9)	4.7 (3.7-5.6)
Missing, No.^c^	54 012	8353	NA	104 842	9690	NA
Education						
<High school	1 461 945 (18.0)	253 704 (15.9)	17.4 (15.3-19.4)	2 426 247 (18.0)	338 315 (15.3)	13.9 (12.4-15.5)
High school or GED	2 896 891 (35.6)	565 893 (35.5)	19.5 (17.9-21.1)	4 755 497 (35.2)	794 116 (35.9)	16.7 (15.4-18.0)
Some college	2 764 662 (34.0)	561 412 (35.2)	20.3 (18.4-22.2)	4 557 562 (33.8)	788 958 (35.7)	17.3 (15.9-18.7)
College grad	1 014 481 (12.5)	213 348 (13.4)	21.0 (18.0-24.1)	1 752 138 (13.0)	290 999 (13.2)	16.6 (14.6-18.6)
Missing, No.^c^	16 461	4507	NA	34 904	5531	NA
Annual household income, $						
<25 000	2 144 972 (31.1)	423 800 (30.6)	19.8 (17.7-21.8)	3 416 114 (30.0)	597 956 (31.2)	17.5 (15.9-19.1)
25 000-49 999	2 056 565 (29.8)	451 581 (32.6)	22.0 (19.7-24.2)	3 312 610 (29.1)	605 717 (31.6)	18.3 (16.7-19.9)
50 000-74 999	1 053 107 (15.3)	200 843 (14.5)	19.1 (17.4-20.8)	1 711 340 (15.0)	259 411 (13.5)	15.2 (13.7-16.6)
75 000-99 999	711 099 (10.3)	135 256 (9.8)	19.0 (16.5-21.5)	1 247 661 (11.0)	206 540 (10.8)	16.6 (13.8-19.3)
≥100 000	928 425 (13.5)	175 742 (12.7)	18.9 (15.7-22.2)	1 688 024 (14.8)	245 290 (12.8)	14.5 (12.1-16.9)
Missing, No.^c^	1 260 271	211 643	NA	2 150 599	303 006	NA
General health						
Excellent	373 096 (4.6)	55 520 (3.5)	14.9 (12.6-17.2)	767 300 (5.7)	86 464 (3.9)	11.3 (9.6-12.9)
Very good	1 679 912 (20.7)	268 603 (16.9)	16.0 (13.8-18.2)	2 952 938 (21.9)	377 234 (17.1)	12.8 (11.3-14.3)
Good	2 861 790 (35.2)	549 967 (34.6)	19.2 (17.1-21.3)	4 715 621 (35.0)	749 331 (33.9)	15.9 (14.3-17.4)
Fair	2 102 973 (25.9)	445 829 (28.0)	21.2 (19.4-23.0)	3 397 316 (25.2)	640 474 (29.0)	18.9 (17.4-20.3)
Poor	1 112 015 (13.7)	271 741 (17.1)	24.4 (21.7-27.2)	1 654 784 (12.3)	356 154 (16.1)	21.5 (19.4-23.7)
Missing, No.^c^	24 653	7206	NA	38 389	8263	NA

Abbreviations: GED, General Educational Development; LCS, lung cancer screening; NA, not applicable; USPSTF, US Preventive Services Task Force.

^a^ Responders were asked if they had ever had a computed tomography (CT) or computerized axial tomography (CAT) scan of the chest area, and if yes, they were asked, “Were any of the CT or CAT scans of your chest area done mainly to check or screen for lung cancer?” Responders who answered yes were then asked, “When did you have your most recent CT or CAT scan of your chest area mainly to check or screening for lung cancer?” Those who responded, “Within the past year (anytime less than 12 months ago)” were classified as having lung cancer screening per recommendations.

^b^ Race and ethnicity in the Center for Disease Control and Prevention Behavioral Risk Factor Surveillance System (BRFSS) are collected via self-report. Self-reported race options on the BRFSS include American Indian or Alaska Native, Asian, Black, Pacific Islander, White, don't know or not sure, and refused. The self-reported ethnicity option on the BRFSS was Hispanic, Latino/a, or Spanish origin. Respondents who reported that they were of more than 1 race group and were not of Hispanic origin were classified as multiracial, non-Hispanic. Race and ethnicity were 2 different questions and were combined so that race responses were not used for those who answered yes to Hispanic/Latino/a or Spanish origin.

^c^ Percentages for the known categories are calculated using the nonmissing population.

We created 2 nested groups by USPSTF eligibility criteria: individuals ages 55 to 79 years with a smoking history of 30 or more PYs or ages 50 to 79 years with 20 or more PYs (2013 and 2021 criteria, respectively). We applied BRFSS survey weights to calculate the weighted number of LCS-eligible and screened individuals, then calculated LCS prevalence rates with 95% CIs in each group by characteristics and state. Data were analyzed using SAS statistical software version 9.4 (SAS Institute) and visualized using R statistical software version 4.3.2 (R Project for Statistical Computing).

## Results

In 2022, the weighted LCS-eligible population was 13 526 348 individuals vs 8 154 440 individuals per 2021 vs 2013 criteria (an increase of 5 371 908 individuals [65.9%]) (Table). The 2022 LCS prevalence was 16.4% and 19.6% using 2021 and 2013 criteria, respectively; the number screened increased by 619 054 individuals. Among individuals newly eligible for LCS under 2021 criteria, 2 063 840 individuals were aged 50 to 54 years, with 6.1% reporting LCS, and 4 020 879 individuals had a 20- to 29-PY smoking history, with 13.1% reporting LCS. Expanded criteria were associated with the greatest relative increase in the LCS-eligible population among Asian (88% increase), Black (109% increase), and Hispanic (86% increase) groups. The number of females eligible for LCS increased by 78% vs 57% for males.

Using 2021 criteria, 2022 LCS prevalence estimates ranged from 8.6% in Wyoming to 28.7% in Rhode Island (Figure). In general, Northeastern and Mid-Atlantic states had higher LCS prevalence rates. There were no significant differences in LCS prevalence rates for 2021 vs 2013 criteria by state or territory, including the District of Columbia (23.9% vs 19.3%), Illinois (17.5% vs 14.6%), Guam (19.1% vs 10.6%), and Virgin Islands (14.1% vs 3.5%).

**Figure.**
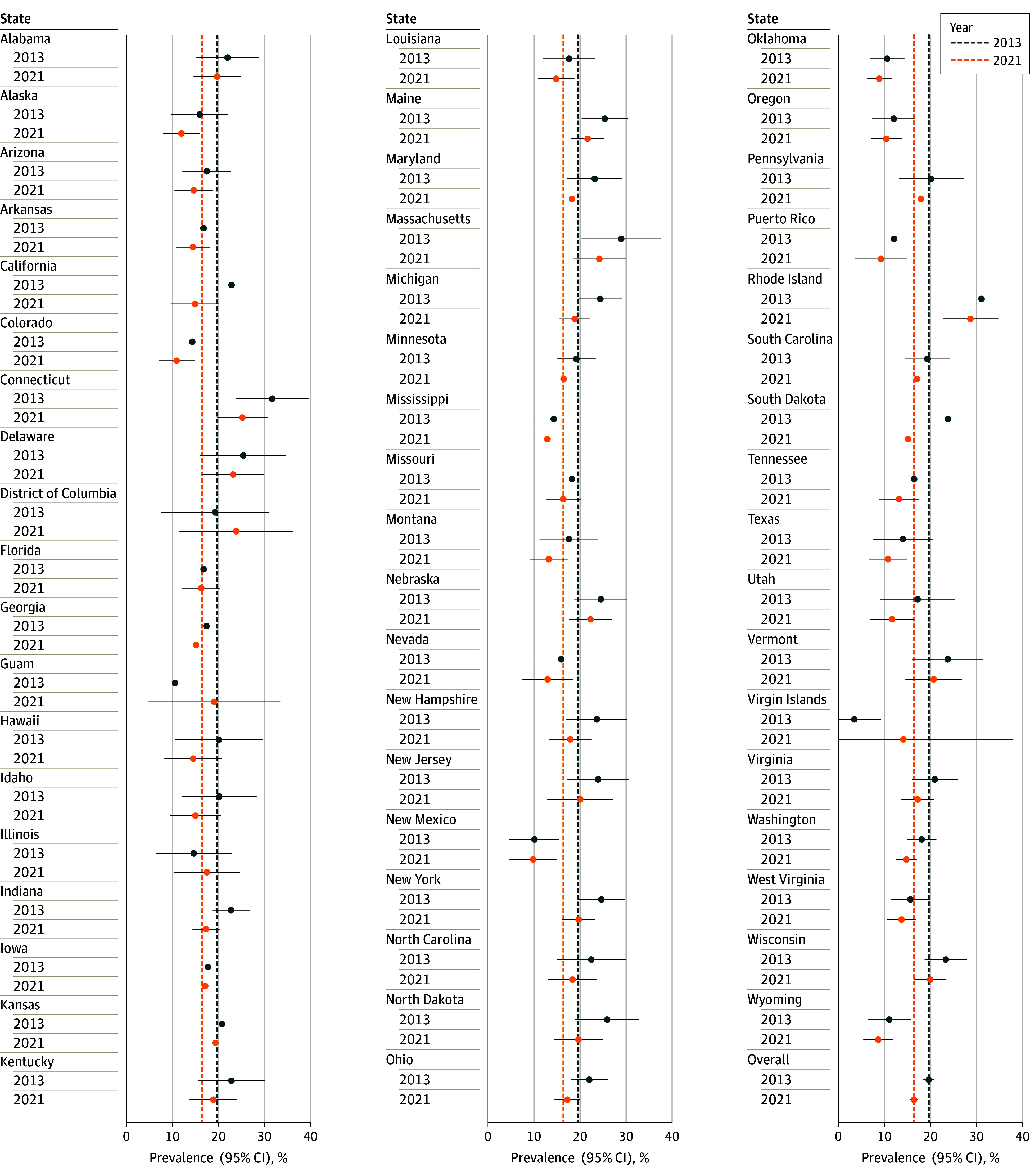
Lung Cancer Screening Prevalence Estimates for 2022 The forest plot presents 2022 lung cancer screening prevalence estimates and 95% CIs for individuals meeting 2013 and 2021 US Preventive Services Task Force eligibility criteria. Vertical lines indicate national 2022 lung cancer screening prevalence estimates using 2013 and 2021 eligibility criteria.

## Discussion

In this cross-sectional study, expanded USPSTF eligibility criteria were associated with 5 371 908 additional individuals eligible for LCS, with relative increases highest for Asian, Black, Hispanic, and female individuals, aligning with the goal of reducing race and ethnic and sex disparities in eligibility.^2,5^ While approximately 619 054 newly eligible individuals were screened under expanded recommendations, 2022 LCS prevalence remained low (16.4%). Prior BRFSS analyses using 2013 USPSTF criteria reported LCS prevalence rates of 12.8% in 2019 (20 states)^6^ and 21.2% in 2021 (4 states),^3^ indicating a similar LCS prevalence rate in 2021 and 2022 (19.6%). A limitation of our study was that BRFSS LCS data are self-reported. Our findings suggest that updated LCS eligibility criteria may be an important first step to reducing lung cancer disparities, although screening rates remained low. Increasing LCS uptake nationwide should be a major public health priority.

## Notes

Supplement.
Data Sharing Statement
