# Supplementary material for: Prevalence of Lung Cancer Screening in the US, 2022
Source: JAMA Netw Open. 2024 Mar 21;7(3):e243190. doi: 10.1001/jamanetworkopen.2024.3190 (PMC10958241; doi:10.1001/jamanetworkopen.2024.3190)
Supplement: Supplement. — Data Sharing Statement [file jamanetwopen-e243190-s001.pdf]

Data Sharing Statement

Henderson. Prevalence of Lung Cancer Screening in the US, 2022. *JAMA Netw Open*. Published online March 21, 2024. doi:10.1001/jamanetworkopen.2024.3190

Data
Data available: Yes
Data types: Other (please specify)
Additional Information: The data used in this study is publicly available and can be downloaded freely.
How to access data: https://www.cdc.gov/brfss/annual_data/annual_data.htm
When available: beginning date: 11-01-2023

Supporting Documents
Document types: None

Additional Information
Who can access the data: These data are publicly available
Types of analyses: n/a
Mechanisms of data availability: freely available
Any additional restrictions: none
